# Effectiveness of Continuous Subcutaneous Insulin Infusion Pump Therapy During Five Years of Treatment on Metabolic Control in Children and Adolescents with Type 1 Diabetes Mellitus

**DOI:** 10.4274/jcrpe.5117

**Published:** 2018-05-18

**Authors:** Özlem Korkmaz, Günay Demir, Hafize Çetin, İlkin Mecidov, Yasemin Atik Altınok, Samim Özen, Şükran Darcan, Damla Gökşen

**Affiliations:** 1 Ege University Faculty of Medicine, Department of Pediatric Endocrinology and Diabetes, İzmir, Turkey

**Keywords:** Type 1 diabetes mellitus, continuous subcutaneous insulin infusion pump therapy, multiple daily insulin therapy, HbA1c

## Abstract

**Objective:**

To compare continuous subcutaneous insulin infusion (CSII) therapy with multiple daily insulin (MDI) therapy on metabolic control in children and adolescents with type 1 diabetes mellitus (T1DM) over the long term.

**Methods:**

Fifty-two T1DM patients treated with CSII and monitored for at least one year prior to and at least five years following CSII were included. Thirty-eight age and sex-matched MDI controls with a 5-year follow up were recruited.

**Results:**

Mean age of the subjects, duration of diabetes and CSII therapy were 17.0±4.8 years, 10.7±2.8 years and 7.7±1.5 years respectively. Mean hemoglobin A1c (HbA1c) in the year prior to CSII, during the first year of treatment and after 5 years of CSII were 7.3±1% (56 mmol/mol), 7.0±0.7% (53 mmol/mol) and 7.8±1.3% (62 mmol/mol) respectively. Initial and 5-year mean HbA1C levels of controls were 7.9±1.08% and 8.6±1.8%. Mean HbA1c values were significantly lower in those receiving CSII therapy throughout follow-up. Basal and total insulin doses were significantly lower in the CSII group at all times. HbA1c was compared between subjects by age (0-5, 6-11 and 12-18 years) with no significant difference between them.

**Conclusion:**

Although CSII mean HbA1c values exceeded accepted good metabolic control limits after 5 years, CSII produces better HbA1c control at all times and in all age groups compared to MDI.

## What is already known on this topic?

With intensive insulin therapy, type 1 diabetic children and adolescents achieve good metabolic control.

## What this study adds?

This data shows that with continuous subcutaneous insulin infusion treatment type 1 diabetic children and adolescents can achieve better metabolic control than multiple daily insulin treatment in the long term.

## Introduction

Continuous subcutaneous insulin infusion (CSII) therapy has been used in the treatment of type 1 diabetes mellitus (T1DM) since the 1970’s and is increasingly used as an alternative to multiple daily insulin (MDI) therapy as pumps have become more widely available. Its effectiveness has been confirmed by meta-analyses of various observational and randomized controlled studies and in childhood and adolescence studies (1,2). The therapeutic goal in T1DM is to establish good and close-to-normal glycemic control, without hypoglycemic attacks, in order to protect against microvascular and macrovascular complications (3). The International Society for Pediatric and Adolescent Diabetes (ISPAD), International Diabetes Federation (IDF) and the American Diabetes Association (ADA) cite a recommended hemoglobin A1c (HbA1c) value of <7.5% in the pediatric age group. 

CSII therapy is the most physiological insulin therapy currently available, more closely mimicking daily insulin release and is also reported to improve patients’ quality of life (4,5). Only a small percentage of patients achieve desired glycemic targets with MDI therapy (4,6). Although several studies have shown a decrease in HbA1c levels with CSII compared to MDI, the HbA1c levels recommended by the ISPAD/IDF/ADA have not been achieved in most studies. In some studies, however, no improvement was observed in HbA1c levels, or levels returned to pre-CSII values at the end of 3-4 years. The limitation of the majority of these studies is the short follow-up time (0.6-8.8 years). Long-term observation studies are therefore needed to determine the efficacy of CSII therapy (7,8,9).

The aim of this study was to assess the effect of CSII treatment on long-term metabolic control in children and adolescents diagnosed with T1DM and compare it to those treated with MDI therapy.

## Methods

Type 1 diabetic patients, aged between 2-18 years, started on CSII therapy between January 2004 and December 2011 at a single centre and subsequently monitored for at least five years were included in the study. Demographic data of the patients, insulin doses, insulin/carbohydrate ratios, daily basal and bolus insulin (food and correction bolus) levels, frequency of capillary blood glucose monitoring, incidence of hypoglycemic attacks, episodes of diabetic ketoacidosis and HbA1c values were obtained retrospectively from file data recorded at every 3-monthly clinic visit. All patients were monitored by a team, consisting of a pediatric endocrinology specialist, a diabetes nurse and a dietician. Anthropometric data were converted to standard deviation scores using Turkish standard data (10). Body mass index (BMI) was calculated using the standard formula weight/height^2 ^(kg/m^2^). HbA1c was measured by turbidimetric inhibition immunoassay (Tina-quant HbA1c Gen. 3) (Normal range: 4.8-5.9%). Severe hypoglycemia was recorded as an event, with symptoms consistent with hypoglycemia in which the patient required assistance from another person or resulted in seizure/coma (11). Incidence rate of severe hypoglycaemic episodes was calculated as number of episodes per 100 patient-years. All children were on Minimed Paradigm Insulin Pump (Minimed Medtronic; Northridge, USA). CSII data for all cases were evaluated with pump data transferred to computer (CareLink**^®^** Pro Therapy Management Software, Minimed Medtronic; Northridge, USA) at each visit. Patients aged between two and 18 years and treated with basal bolus regimen with MDI, with both the same duration of diabetes and a monitoring period of at least five years were enrolled as the control group. CSII patients were classified into three different groups: preschoolers (≤6 years old, n=16), prepubertal (six years to Tanner stage 2, n=18) and pubertal (n=18). Patients who had at least Tanner 2 breast development or testicular volume ≥4 mL were included in the pubertal group (12). CSII patients were also stratified according to good (n=37), moderate (n=9) and poor metabolic control (n=6) which was defined as: HbA1c: <7.5%, 7.5-9% and >9% or <58, 58-75 and >75 mmol/mol, respectively. 

Before initiating CSII therapy, all patients and their families completed a training program. Patients declining informed voluntary consent, with diagnosed psychiatric disorders or a monitoring period of less than five years were excluded from the study. Ege University Medical Faculty Clinical Investigations Ethical committee approval was obtained for the study (no:16-6.1/13).

### Statistical Analysis

Normal distribution of data was assessed using the Shapiro-Wilks test with p>0.05 indicating normal distribution. Chi square analysis was performed for categoric variables. Two-group HbA1c comparisons were performed using the Mann-Whitney test. The Wilcoxon matched two samples test was used to determine variation over time in the groups. T test was used for comparisons between independent variables when comparing CSII and MDI groups. Analysis of variance was performed for recurring measurements in the analysis of groups determined on the basis of age groups. The Bonferroni test was used for multiple comparisons between times. Linear correlation between variables was evaluated using Pearson’s correlation analysis.

## Results

### Demographic Data

Ninety cases diagnosed with type 1 DM were included in the study. Fifty-two patients (57%) were on CSII therapy and 38 (43%) were on MDI. 48.1% were male and 51.9% were female in the CSII group whilst 44.7% were male and 55.3% were female in the MDI group. Mean age and duration of diabetes in the CSII group at the time of enrolment was 17.0±4.8 and 10.7±2.8 years respectively. Mean duration of CSII therapy was 7.7±1.5 years. Mean age of the subjects on MDI therapy was 17.6±3.5 years and duration of diabetes was 10.1±3.9 years There was no statistical difference in terms of age of the subjects and duration of diabetes between the CSII and MDI groups.

### Metabolic Control

**Figure 1:**
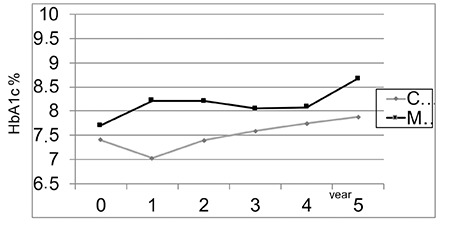
Comparison of the mean hemoglobin A1c values between the two treatment groups during the five year follow-up period HbA1c: hemoglobin A1c

Mean HbA1c in the year prior to initiation of CSII was 7.3±1.0% (56 mmol/mol) while mean HbA1c at the end of the first year of CSII was 7.0±0.7% (53 mmol/mol). At the latter time point HbA1c levels were <7.5% (<58 mmol/mol) in 78.8% of cases. Mean HbA1c at the end of five years was 7.8±1.3% (62 mmol/mol). In the CSII group 19 patients (39%) still had a mean HbA1c <7.5% (<58 mmol/mol) at the end of the fifth year. Mean initial and 5-year HbA1c levels of cases on MDI therapy were 7.7±1.04% (61 mmol/mol) and 8.6±1.8% (70 mmol/mol) respectively and nine (23%) of the patients’ HbA1c were <7.5% (<58 mmol/mol) at the end of fifth year. Mean change in HbA1c at the end of five years in the CSII group was 0.5±1.5% compared with 0.6±1.9% in the MDI group and there was no significant difference between the groups with respect to change in HbA1c (Table 1). Mean HbA1c was significantly lower in the CSII group throughout the five years follow up (p<0.05; Figure 1). No correlation was found between HbA1c levels and age, sex, duration of diabetes, duration of CSII or insulin doses used.

**Figure 2:**
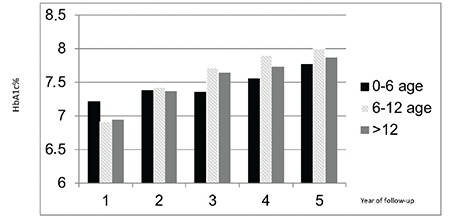
Mean hemoglobin A1c levels in the continuous subcutaneous insulin infusion patients by age group during the five year follow-up period HbA1c: hemoglobin A1c

There was no significant difference in HbA1c in the CSII group when sub-grouped according to age (Figure 2). HbA1c levels increased during follow-up in all age groups.

At the end of the fifth year of CSII therapy, HbA1c of the patients in the well-controlled group (n=37) increased to 7.6±0.8% (60 mmol/mol) from 6.8±0.6% (51 mmol/mol). The group with moderate control (n=9) decreased HbA1c levels during the first year but HbA1c increased by the end of the fifth year. In the poor metabolic control group (n=6), although HbA1c decreased in the first year, at the end of the fifth year it had again increased but in no patient did it exceed pre-treatment HbA1c levels (Table 2).

### Insulin Dosage

**Figure 3:**
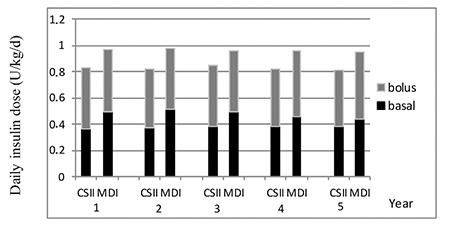
Comparison of the daily insulin dose between the two treatment groups during the five year follow-up period CSII: continuous subcutaneous insulin infusion, MDI: multiple daily insulin

Children using MDI therapy used lower total daily insulin doses compared to those treated with CSII at the beginning of therapy (MDI: 0.96±0.21 U/kg/d; CSII: 1±0.35 U/kg/d respectively). Daily insulin dose decreased to 0.83±0.21 U/kg/d at the end of one year of CSII. No time-dependent changes in daily insulin dose were observed between the two groups during the subsequent years (Figure 3).

Basal insulin dose in the first year of treatment was 0.36±0.14 U/kg/d in the CSII group vs 0.48±0.19 U/kg/d in the MDI group. No time-dependent change was found in basal insulin throughout follow up in the two groups. Basal insulin dose was significantly lower in the CSII group compared to the MDI group in all of the time periods (p<0.05; Figure 3). When cases were stratified by age within the CSII group, the basal insulin doses used by subjects aged over 12 years was higher than that in the other age groups. 

Bolus insulin used in the first year of treatment was 0.46±0.25 U/kg/d in the CSII group and 0.47±0.17 U/kg/d in the MDI group. No statistically significant difference was found between the groups at the 5-year follow period (Figure 3). Neither was there a difference in terms of bolus insulin doses found between age groups.

### Anthropometric Data

**Figure 4:**
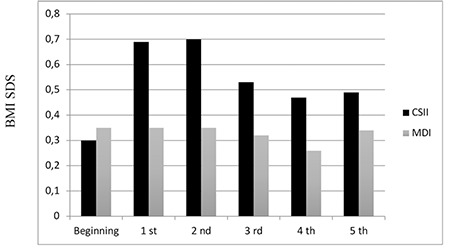
Body mass index standard deviation score values during the five-year follow-up period CSII: continuous subcutaneous insulin infusion, MDI: multiple daily insulin, BMI: body mass score, SDS: standard deviation score

BMI SDS at start of therapy was 0.39±0.95 SD in the CSII group and 0.39±0.85 SD in the MDI group. At the end of five years it was 0.49±1.01 SD in the CSII group and 0.34±0.87 SD in the MDI group. At the beginning of the study, there were three obese cases, but this increased to five at the end of the study period (two CSII, three MDI). Although BMI SDS in the CSII group increased in the first and second years, there was no statistically significant difference between the groups at 5-year follow up (Figure 4).

### Adverse Events

Diabetic ketoacidosis was observed in one of the CSII cases (0.31/100-patient-years) and four of the patients on MDI treatment (2.1/100-patient-years) during monitoring, while severe hypoglycemia was seen in two patients in the CSII group (0.62/100-patient-years) and in one case in the MDI group (1.9/100-patient-years).

## Discussion

CSII is a safe and effective therapeutic technique in children and adolescents diagnosed with type 1 DM. There has been a significant increase in its use in the last 10 years, although there are still differences in rates of use between countries (13). Various studies have shown that CSII improves glycemic control and increases patients’ quality of life, without increasing the incidence of hypo or hyperglycemic episodes (14,15,16). However, target HbA1c levels of <7.5%, based on ADA/IDF/ISPAD recommendations, have not been achieved in the majority of these studies. Although there have been several studies investigating the effectiveness of CSII, a short monitoring period has been a limitation in most of these studies (4). In one study over a two year follow up period, HbA1c levels, despite improving in the first six months, tended to increase over the subsequent 18-month period in pump patients (17). At longer term follow-up of up to five years, the initial decrease in HbA1c was described as a “temporary improvement”, while an increase in HbA1c levels was observed in later periods (17). A meta-analysis of results of various randomized, controlled studies has shown that a decrease in HbA1c levels of 0-0.9% has been achieved with CSII when the duration of intervention ranged from six to 12 months (18). At the end of the first year in this analysis there was a significant decrease in HbA1c with 78.8% of the patients <7.5% (58 mmol/mol). Although mean HbA1c levels were lower in the patients receiving CSII during follow up, mean HbA1c increased to 7.8±1.3% (62 mmol/mol) at the end of the fifth year from 7.3±1.0% (56 mmol/mol) before CSII initiation. The increase in HbA1c in our patients after the first year could be due to increasing age, duration of diabetes or due to decreased compliance of the patients. One multi-center study reported lower HbA1c levels in all age groups in a group receiving CSII compared to MDI patients (13). Another study from Denmark showed lower HbA1c levels at all years in the CSII group, followed up for more than five years, in keeping with our findings (19). In the Danish study, although a marked improvement was observed in HbA1c levels in the first year of CSII, HbA1c levels tended to increase in subsequent years with the best metabolic control established one year after CSII initiation. We found exactly the same pattern of metabolic control in our study group. The lower mean HbA1c in the CSII group throughout the five years may be due to short duration of CSII treatment, which is a relatively recent treatment modality, compared to MDI treatment. 

At the end of fifth year of therapy, HbA1c of the patients in the good control and moderate control groups increased. In the poorly controlled group, HbA1c decreased in the first year similarly to the other groups but the rate of increase after the first year was slower than the other groups. A future therapeutic aim should be to develop new approaches to prevent the impairment of metabolic control over the long-term, so that the short-term improvements seen in metabolic control in first year of therapy might be maintained. Repetition of periodic diabetes education and planning of practices that increase motivation, such as motivational interviewing, may be helpful and should be investigated. 

When metabolic control was analyzed according to age groups no significant difference was observed throughout the five-year follow-up period between the age groups in our study. In a study by Johnson et al (20), with respect to different age groups, the older age groups (two groups; 6-12 years and >12 years) had the most dramatic initial improvement of glycemic control, compared with the youngest subject (<6-years) group upon commencement of insulin pump therapy, with HbA1c decreasing by 0.6 to 0.8% within three months. Over the following five years, each age group on CSII showed an improvement compared with non-CSII counterparts. However, the initial HbA1c was lowest in the <6-year-old group, followed by the six to 12 year olds and then the >12 year olds. The mean HbA1c of the <6-year-old pump cohort remained below 7.5% (58 mmol/mol) from six months through the first five years of follow-up.

An increase was found in BMI SDS in the first and second years of CSII in our study. This might be attributed to patients initially adopting a more flexible dietary model with CSII. However, at the end of 5-year follow-up, no significant difference was seen between the CSII and MDI groups in terms of mean BMI SDS. There was no relation between BMI SDS and poor metabolic control. The SWEET study group reported similar BMI SDS in CSII and MDI patients (13). Significant increase in BMI SDS was found in the 6-12 age group when compared with the MDI treatment group, but when linear regression analysis was performed on the basis of duration of diabetes, no significant variation was observed between the two treatment groups. Johnson et al (20) also reported a similar change in BMI SDS in CSII and MDI groups. No difference was found in change in BMI SDS during follow-up in terms of the age groups in our study (p=0.885). There was also no correlation between HbA1c and BMI SDS. The impact of CSII treatment on BMI varies in the literature without any clear pattern emerging. Therefore longer follow-up periods may be helpful in drawing a conclusion on BMI in children and adolescents on pump therapy.

Total daily insulin doses recommended by IDF/ISPAD in prepubertal and pubertal children are 0.7-1U/kg/d and 1-2U/kg/d, respectively. A potentially greater insulin requirement has been reported due to insulin resistance in puberty (21). In our study, total insulin dose used before initiation of CSII was significantly higher than the total dose used after CSII therapy commencement and the MDI group’s total daily insulin dose was higher than the CSII group throughout follow up. In the SWEET study CSII patients used lower-dose insulin compared to subjects on MDI (13). Similarly, Pickup et al (1) also observed a lower daily insulin dose in the CSII group. When patients in our study were analyzed according to age groups, daily and basal insulin doses were higher in subjects over 12 years, possibly reflecting early puberty insulin requirement increases or to a more flexible life style change with CSII, or a combination of the two factors. It has been reported that lower HbA1c levels are associated with higher basal insulin levels (22). However, no relation between HbA1c and basal insulin doses was observed in our study.

### Study Limitation

The main limitation of this study is the retrospective design of data from a single center data. In addition, the frequency of patients’ hypo- or hyperglycemic episodes, other than the most severe ones, are not included in the study. Conversely as a single center study, all the patients were monitored with the same treatment protocol which is a strength in terms of standardization of laboratory results, measurement techniques, patient counseling and team approach to management.

## Conclusion

Although HbA1c values were not within recommended metabolic control limits with either treatment modality at the end of the five years follow up, CSII produces better metabolic control compared to MDI over the long-term.

## Figures and Tables

**Table 1:**
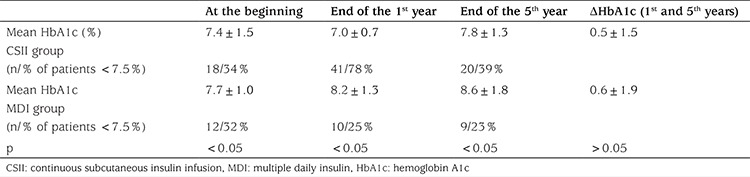
Comparison of metabolic control during the five year follow up in continuous subcutaneous insulin infusion and multiple daily insulin groups

**Table 2:**
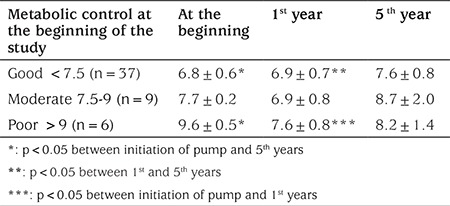
Comparison of metabolic control during the five year follow-up in the continuous subcutaneous insulin infusion group in terms of good, moderate and poor metabolic control prior to pump therapy initiation
